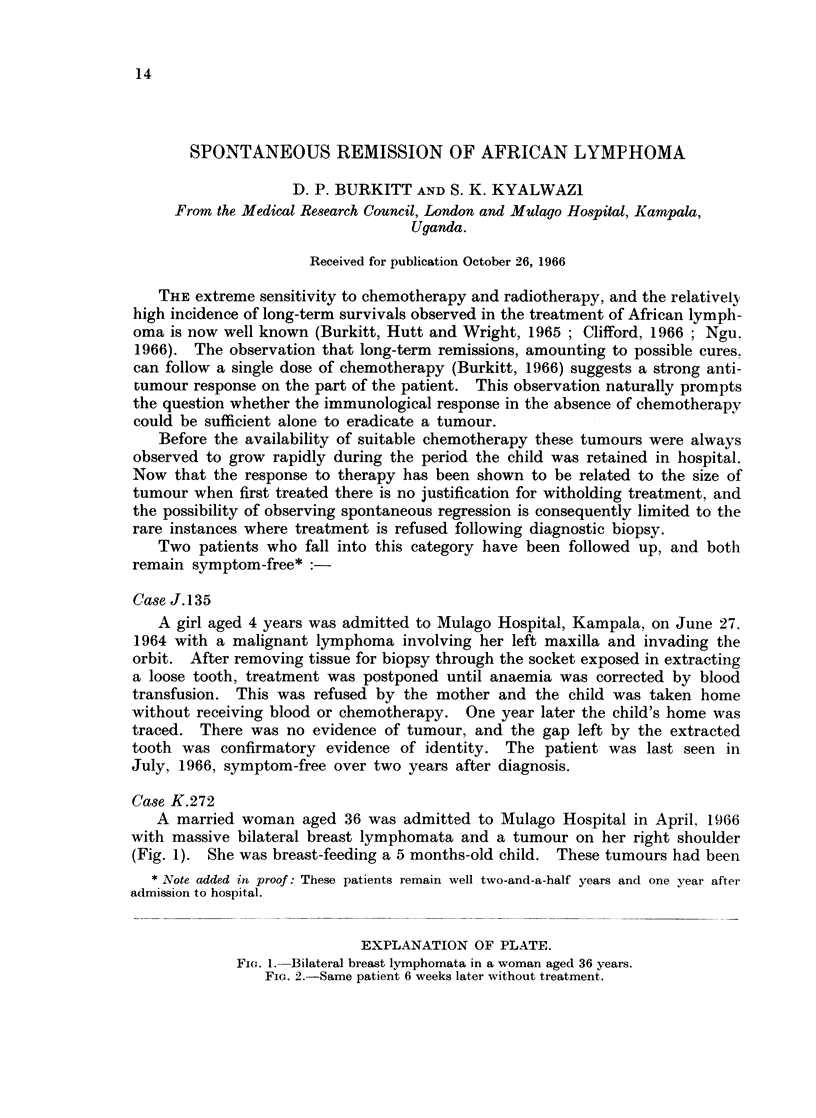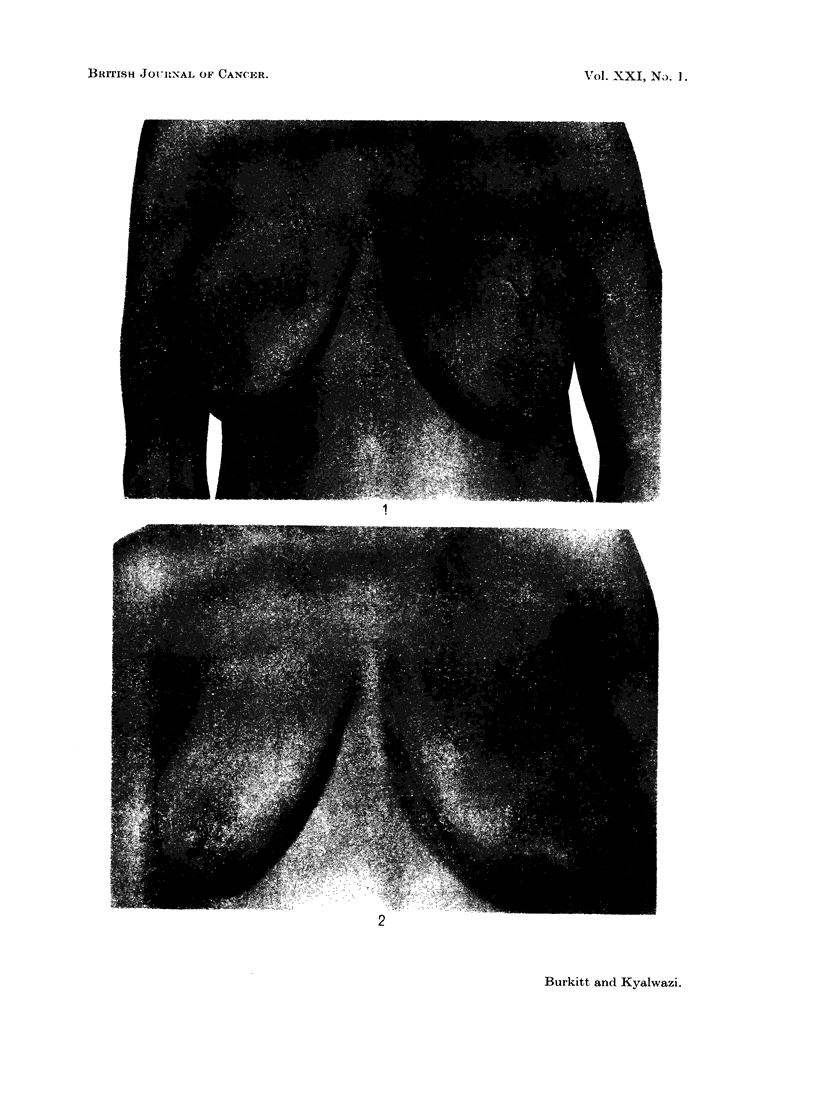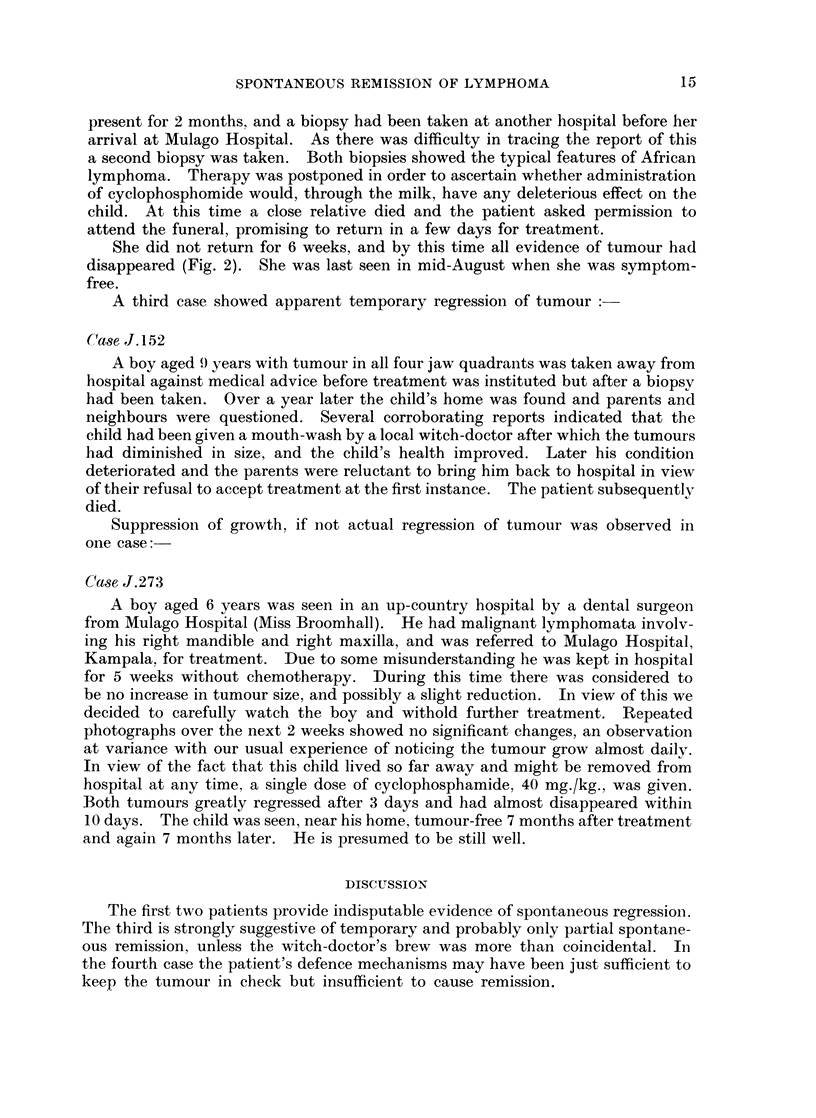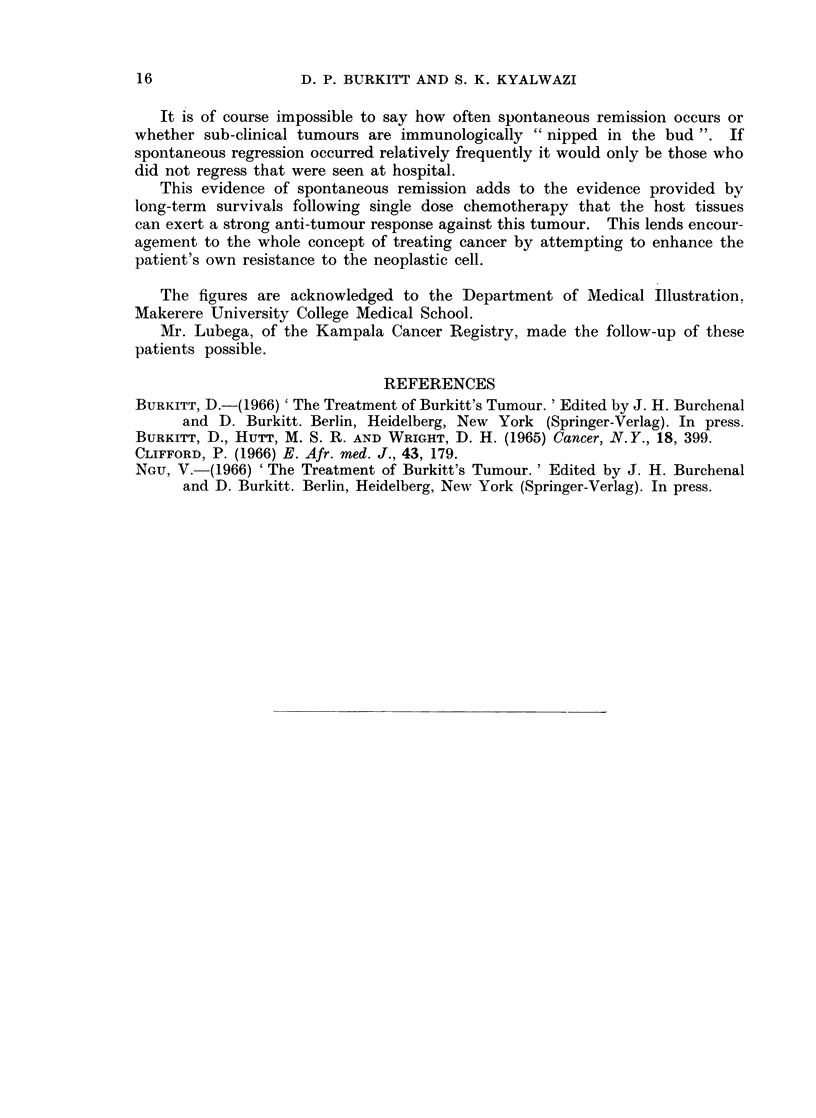# Spontaneous remission of African lymphoma.

**DOI:** 10.1038/bjc.1967.2

**Published:** 1967-03

**Authors:** D. P. Burkitt, S. K. Kyalwazi

## Abstract

**Images:**


### SPONTANEOUS REMISSION OF AFRICAN LYMPHOMA

D. P. BURKITT AND S. K. KYALWAZI
*From the Medical Research Council, London and Mulago Hospital, Kampala, Uganda.*

Received for publication October 26, 1966

THE extreme sensitivity to chemotherapy and radiotherapy, and the relatively high incidence of long-term survivals observed in the treatment of African lymphoma is now well known (Burkitt, Hutt and Wright, 1965 ; Clifford, 1966 ; Ngu, 1966). The observation that long-term remissions, amounting to possible cures, can follow a single dose of chemotherapy (Burkitt, 1966) suggests a strong anti-tumour response on the part of the patient. This observation naturally prompts the question whether the immunological response in the absence of chemotherapy could be sufficient alone to eradicate a tumour.

Before the availability of suitable chemotherapy these tumours were always observed to grow rapidly during the period the child was retained in hospital. Now that the response to therapy has been shown to be related to the size of tumour when first treated there is no justification for witholding treatment, and the possibility of observing spontaneous regression is consequently limited to the rare instances where treatment is refused following diagnostic biopsy.

Two patients who fall into this category have been followed up, and both remain symptom-free* :—

*Case J.*135

A girl aged 4 years was admitted to Mulago Hospital, Kampala, on June 27, 1964 with a malignant lymphoma involving her left maxilla and invading the orbit. After removing tissue for biopsy through the socket exposed in extracting a loose tooth, treatment was postponed until anaemia was corrected by blood transfusion. This was refused by the mother and the child was taken home without receiving blood or chemotherapy. One year later the child's home was traced. There was no evidence of tumour, and the gap left by the extracted tooth was confirmatory evidence of identity. The patient was last seen in July, 1966, symptom-free over two years after diagnosis.

*Case K.*272

A married woman aged 36 was admitted to Mulago Hospital in April, 1966 with massive bilateral breast lymphomata and a tumour on her right shoulder (Fig. 1). She was breast-feeding a 5 months-old child. These tumours had been

* *Note added in proof:* These patients remain well two-and-a-half years and one year after admission to hospital.

---

EXPLANATION OF PLATE.

FIG. 1.—Bilateral breast lymphomata in a woman aged 36 years.
FIG. 2.—Same patient 6 weeks later without treatment.

1

2

Burkitt and Kyalwazi.

present for 2 months, and a biopsy had been taken at another hospital before her arrival at Mulago Hospital. As there was difficulty in tracing the report of this a second biopsy was taken. Both biopsies showed the typical features of African lymphoma. Therapy was postponed in order to ascertain whether administration of cyclophosphomide would, through the milk, have any deleterious effect on the child. At this time a close relative died and the patient asked permission to attend the funeral, promising to return in a few days for treatment.

She did not return for 6 weeks, and by this time all evidence of tumour had disappeared (Fig. 2). She was last seen in mid-August when she was symptom-free.

A third case showed apparent temporary regression of tumour :—

*Case J.*152

A boy aged 9 years with tumour in all four jaw quadrants was taken away from hospital against medical advice before treatment was instituted but after a biopsy had been taken. Over a year later the child's home was found and parents and neighbours were questioned. Several corroborating reports indicated that the child had been given a mouth-wash by a local witch-doctor after which the tumours had diminished in size, and the child's health improved. Later his condition deteriorated and the parents were reluctant to bring him back to hospital in view of their refusal to accept treatment at the first instance. The patient subsequently died.

Suppression of growth, if not actual regression of tumour was observed in one case:—

*Case J.*273

A boy aged 6 years was seen in an up-country hospital by a dental surgeon from Mulago Hospital (Miss Broomhall). He had malignant lymphomata involving his right mandible and right maxilla, and was referred to Mulago Hospital, Kampala, for treatment. Due to some misunderstanding he was kept in hospital for 5 weeks without chemotherapy. During this time there was considered to be no increase in tumour size, and possibly a slight reduction. In view of this we decided to carefully watch the boy and withold further treatment. Repeated photographs over the next 2 weeks showed no significant changes, an observation at variance with our usual experience of noticing the tumour grow almost daily. In view of the fact that this child lived so far away and might be removed from hospital at any time, a single dose of cyclophosphamide, 40 mg./kg., was given. Both tumours greatly regressed after 3 days and had almost disappeared within 10 days. The child was seen, near his home, tumour-free 7 months after treatment and again 7 months later. He is presumed to be still well.

### DISCUSSION

The first two patients provide indisputable evidence of spontaneous regression. The third is strongly suggestive of temporary and probably only partial spontaneous remission, unless the witch-doctor's brew was more than coincidental. In the fourth case the patient's defence mechanisms may have been just sufficient to keep the tumour in check but insufficient to cause remission.

It is of course impossible to say how often spontaneous remission occurs or whether sub-clinical tumours are immunologically " nipped in the bud ". If spontaneous regression occurred relatively frequently it would only be those who did not regress that were seen at hospital.

This evidence of spontaneous remission adds to the evidence provided by long-term survivals following single dose chemotherapy that the host tissues can exert a strong anti-tumour response against this tumour. This lends encouragement to the whole concept of treating cancer by attempting to enhance the patient's own resistance to the neoplastic cell.

The figures are acknowledged to the Department of Medical Illustration, Makerere University College Medical School.

Mr. Lubega, of the Kampala Cancer Registry, made the follow-up of these patients possible.

### REFERENCES

BURKITT, D.—(1966) ' The Treatment of Burkitt's Tumour. ' Edited by J. H. Burchenal and D. Burkitt. Berlin, Heidelberg, New York (Springer-Verlag). In press.

BURKITT, D., HUTT, M. S. R. AND WRIGHT, D. H. (1965) *Cancer, N.Y.*, **18**, 399.

CLIFFORD, P. (1966) *E. Afr. med. J.*, **43**, 179.

NGU, V.—(1966) ' The Treatment of Burkitt's Tumour. ' Edited by J. H. Burchenal and D. Burkitt. Berlin, Heidelberg, New York (Springer-Verlag). In press.